# Vascular cognitive impairment

**Published:** 2009-01

**Authors:** Alladi Suvarna

**Affiliations:** Department of Neurology, Nizam’s Institute of Medical Sciences, Hyderabad - 500 082, India

**Keywords:** Dementia, vascular cognitive impairment, stroke, vascular dementia, vascular risk factors

## Abstract

The term vascular cognitive impairment (VCI) has been proposed to encompass all people with cognitive impairment of cerebrovascular origin. VCI is not a single condition, but has several clinical presentations, etiologies, and treatment. VCI forms a spectrum that includes vascular dementia, mixed Alzheimer’s disease with a vascular component, and VCI that does not meet dementia criteria. Multiple pathophysiological mechanisms contribute to VCI, accounting for its heterogeneity. Although main changes in the brain in VCI include cerebral infarcts, vascular cognitive impairment is thought to be due to factors beyond acute infarcts. Cerebral white matter lesions and silent brain infarcts are considered to be risk factors for VCI. The prevalence of VCI is high and this entity is poised to become the silent epidemic of the 21st century. Cognitive impairment due to cerebrovascular disease can to some extent be improved, and VCI prevented, if vascular risk factors are brought under control and strokes do not recur. Therefore, strategies that focus on the prevention and treatment of the cognitive impairment associated with cerebrovascular disease are high priority healthcare objectives.

## INTRODUCTION

Cognitive impairment that is caused by or associated with vascular factors has been termed “vascular cognitive impairment” (VCI).[1] VCI is not a single condition, but has several clinical presentations, etiologies, and treatment. There is increasing evidence that patients with clinically significant cognitive impairment in association with vascular disease frequently do not fulfill the traditional criteria of dementia.[2] As the condition is preventable to a large extent, it is important to identify patients at early stages of cognitive impairment, to treat appropriately and prevent progression to frank dementia.[1]

## MAGNITUDE OF VCI

Studies have shown that up to 64% of persons who have experienced a stroke have some degree of cognitive impairment[3] with up to a third developing frank dementia.[4] Conversely, postmortem pathological studies indicate that up to 34% of dementia cases show significant vascular pathology.[5] VCI is therefore poised to become the silent epidemic of the 21st century.[6] Vascular dementia (VaD) is the second most common cause of dementia after Alzheimer disease (AD), and in some Asian countries, it is the most frequent cause.[6,7] The prevalence of VaD ranges from 1 to 8.8%.[2,8] An epidemiological study from India found VaD to account for 39% of dementias compared with 54% of AD.[9] Post-stroke dementia, affects about 30% of patients older than 65 years after ischemic stroke.[10] In a hospital based stroke clinic in South India, 75 consecutive stroke patients were evaluated for cognitive impairment three months after stroke. Post stroke dementia was detected in 20 (26.6%), milder form of cognitive impairment in 20 (26.6%) and no cognitive impairment in 25 patients (33%) using a standard neuropsychological battery of tests.[11]

Cardiovascular (CV) risk factors that include diabetes, hypertension, hyperlipidemia, and smoking are highly prevalent and cardiovascular diseases contribute significantly to mortality and disability.[12] There is increasing evidence from India and other countries that CV risk factors are associated with an increased risk of cognitive decline and dementia.[13,14] Cognitive impairment due to cerebrovascular disease can to some extent be improved, and VCI prevented, if vascular risk factors are brought under control and strokes do not recur.[15] Therefore strategies that focus on the prevention and treatment of the cognitive impairment associated with cerebrovascular disease are high priority healthcare objectives.

## PATHOPHYSIOLOGY

Multiple pathophysiological mechanisms contribute to VCI, accounting for its heterogeneity. Although main changes in the brain in VCI include cerebral infarcts, VCI is thought to be due to factors beyond acute infarcts.[16] Cerebral white matter lesions (WML) and silent brain infarcts are considered to be risk factors for dementia.[17,18] Large artery disease, small artery disease, cardioembolism are important underlying causes of VCI. Vascular mechanisms underlying VCI are influenced by the pattern of stroke mechanisms, which differ among Asian countries compared with the west; small artery and intracranial large artery disease contribute to a larger proportion of underlying vascular mechanisms in India.[19,20] Increasing evidence suggests that hippocampal and cerebral atrophy is associated with VCI.

## CLINICAL SPECTRUM OF VCI

VCI forms a spectrum that includes VaD, mixed AD with a vascular component, and VCI that does not meet dementia criteria. In a hospital based registry in South India, of 87 patients with VCI, 59.8% had VaD, 31% had cognitive impairment without dementia and 9.2% had mixed dementia.[20] VaD is a group of syndromes that represent a clinicoradiologic and pathological spectrum. Three subtypes recognizable in clinical practice include, multi infarct dementia characterized by recurrent stroke, stepwise course, focal neurological symptoms and signs and multiple cerebral infarcts on brain imaging, strategic infarct dementia characterized by an abrupt onset of memory impairment or behavioral change in association with a single, strategically placed infarct, and subcortical vascular dementia due to small-vessel disease, which leads to white matter and deep subcortical gray and white matter demyelination and lacunar infarcts.[21] A hospital based study in India, characterizing pattern of VaD diagnosed according to Neurological Disorders and Stroke (NINDS) AIREN criteria demonstrated that subcortical dementia was the most common form of VaD, followed by cortical-subcortical dementia.[19]

In comparison to AD, there is a general consensus that episodic memory is more impaired in AD, and that executive/attentional processing is more impaired in vascular dementia, especially in patients with subcortical VaD.[22] Some recent data suggest a less exclusive role for executive dysfunction in VCI than was previously proposed.[23] Patients with VaD also showed greater impairment in both semantic memory and visuospatial/perceptual function than the patients with Alzheimer’s disease.[24] Further, depressive symptoms were more common in patients with VCI.[25]

Recent evidence indicates a complex interaction between VaD and AD. Longitudinal studies have shown that vascular risk factors pre-dispose to both cerebrovascular disease and AD.[26] Further, considerable overlap between vascular and AD pathology exists in dementia patients.[27] Diffuse neurochemical abnormalities, especially cholinergic deficits have been identified in VaD. Furthermore, brain vascular and neurodegenerative pathologies may be additive in the way in which they influence clinical presentation.[7] A population based autopsy study found pure VaD in 13% of dementias and mixed VaD and AD in 12% in comparison to 51% of AD.[5] The results suggest that clinical diagnosis of dementia made during life may fail to reflect the pathogenic complexity of this condition in elderly persons.

## VCI HARMONIZATION STANDARDS

A uniform cognitive, clinical, imaging and pathological profile typical of VCI has not been identified. The reasons are partly methodological and also due to heterogeneity of patient groups in studies. In an attempt to identify and describe individuals with VCI, particularly in the early stages, The National Institute for NINDS and the Canadian Stroke Network (CSN) developed common standards in clinical diagnosis, epidemiology, brain imaging, neuropathology, experimental models, genetics, and clinical trials to recommend minimum, common, clinical and research standards for the description and study of VCI.[28] Using the same standards was thought to help identify individuals in the early stages of cognitive impairment, make studies comparable, and integrate knowledge, thereby accelerating the pace of progress of understanding, preventing and treating VCI.

## COURSE AND OUTCOME OF DISEASE

Nearly half of elderly with mild cognitive impairment (MCI) due to vascular disease converted to dementia after five years in the Canadian Study of Health and Aging.[29]

Further, a follow up study of people with VCI, AD, and ‘No cognitive impairment,’ showed that most people with VCI showed readily detectable progression by 30 months and depressive symptoms and impaired judgment progressed more commonly in patients with VCI.[25] In a longitudinal study of VaD due to subcortical lacunar infarcts, progressive cognitive decline was determined mainly by the occurrence of new vascular episodes and severity of the cognitive impairment at baseline, providing evidence that ongoing vascular insult is responsible for progression of disease.[30] Vascular dementia shortens life expectancy. A 5-year follow-up study of incident dementia cases[31] found the mortality risk was 3.3 times higher for vascular dementia compared with non-demented people.

## TREATMENT AND PREVENTION

Attempts to reverse or delay progression of cognitive impairment due to cerebrovascular disease have given grounds for hope of treatment of VAD. Based on the various pathophysiological mechanisms proposed to underlie VaD, a wide range of drugs have been tried to treat VaD.

In a randomized, trial of 325 mg/day aspirin (vs. no aspirin) conducted on multi-infarct dementia patients. The group of aspirin patients showed significantly higher cognitive scores than the untreated group.[32] The use of acetylcholinesterase inhibitors is based on the demonstration of existence of cholinergic deficits in pure VaD. Donepezil tested in two double-blind, placebo-controlled trials on patients diagnosed with possible or probable VaD showed significant improvement in cognition (ADAS-cog, MMSE) and global functioning scores.[33] Galantamine, rivastigmine, and memantine have also demonstrated improvement in cognition and caregiver stress. Pentoxifylline, propentofylline, citicholine, hydergine, nicergoline, and piracetam, were found to have a positive effect on cognitive function and behavior.

The prevention of stroke recurrence through the control of risk factors, by carotid endarterectomy as well as the use of antithrombotic drugs (warfarin or antiplatelet agents), is likely to reduce the incidence of VaD and mixed dementia. Decreased incidence of dementia associated with antihypertensive treatment using candesartan, nitrendipine and perindopril has been demonstrated.[34,35] Some studies suggest that the use of lipid-lowering agents lowers the risk of dementia and protects against cognitive decline.[36]

## CONCLUSIONS

Vascular risk factors and cerebrovascular disease are now recognized to account for a major proportion of cognitive disorders, including degenerative dementias. Emphasis has shifted from diagnosis of VaD based on restrictive criteria to include a broader spectrum of disease VCI that includes mixed dementia and cognitive impairment with no dementia. Control of vascular risk factors and treatment of milder forms of disease need to form the focus of preventive strategies.
